# Use of the Human Vaccine, *Mycobacterium bovis* Bacillus Calmette Guérin in Deer

**DOI:** 10.3389/fvets.2018.00244

**Published:** 2018-10-08

**Authors:** Mitchell V. Palmer, Tyler C. Thacker

**Affiliations:** Infectious Bacterial Diseases of Livestock Research Unit, National Animal Disease Center, Agricultural Research Service, United States Department of Agriculture, Ames, IA, United States

**Keywords:** BCG, deer, mycobacterium, tuberculosis, vaccine, wildlife

## Abstract

The only vaccine ever approved for human tuberculosis was developed a century ago from an isolate of *Mycobacterium bovis* derived from a tuberculous cow. Initial safety and efficacy studies of an attenuated version of this isolate were conducted in cattle and other animals. In 1921 the first human, an infant, was orally dosed with this attenuated strain that came to be known as *M. bovis* bacillus Calmette-Guérin (BCG); named for Albert Calmette and Camille Guérin, the two French scientists that developed the strain. Since 1921, billions of people have been vaccinated with BCG making it the oldest, most widely used, and safest vaccine in use today. It is also the tuberculosis vaccine most studied for use in wildlife, including deer. While BCG vaccination of deer may not reliably prevent infection, it consistently decreases lesion severity, minimizing large, necrotic lesions, which often contain large numbers of bacilli. It is believed that decreased lesion severity correlates with decreased disease transmission; however, this hypothesis remains to be proven. Safety studies in white-tailed deer show BCG may persist in lymphoid tissues for up to 12 months; a factor to be considered in deer used for food. Beyond efficacy and safety, methods of vaccine delivery to free-ranging deer are also under investigation, both in the laboratory and in the field. The ideal delivery method is effective, efficient and safe for non-target species, including livestock. Ingestion of BCG by cattle is of special concern as such cattle may present as “false positives” using currently approved diagnostic methods, thus interfering with efforts by animal health agencies to monitor cattle for tuberculosis. An effective BCG vaccine for deer would be of value in regions where free-ranging deer represent a potential source of *M. bovis* for livestock. Such a vaccine would also be beneficial to farmed deer where *M. bovis* represents a serious threat to trade and productivity.

## Introduction

*Mycobacterium bovis* is the cause of tuberculosis in most animal species, including man. Clinical signs and pathological manifestations of *M. bovis* in humans can be identical to infection with the more common cause of human tuberculosis, *Mycobacterium tuberculosis*. The range of susceptible hosts to *M. bovis* is broad and includes most species of both livestock and wildlife. For decades, most developed countries have conducted costly campaigns to eradicate tuberculosis from cattle with varying success (1). In cases where a wildlife reservoir of *M. bovis* infection exists, eradication has been difficult, if not impossible (2) due to transmission of *M. bovis* from livestock to wildlife (spillover) and subsequent transmission from wildlife back to livestock (spillback). In northeast Michigan, USA there is a focus of *M. bovis* infection in free-ranging white-tailed deer (*Odocoileus virginianus*) where infected deer have been implicated as the source of infection in 69 cattle herds from 1995 through 2017. Control efforts, including increased hunting have been effective in decreasing disease prevalence from 4.9% in 1995 to 1.7% in 2004, but prevalence continues to remain at approximately 2% (3, 4).

In addition to white-tailed deer in the US, there is general consensus that the European badger (*Meles meles*) in the United Kingdom and the Republic of Ireland, the brushtail possum (*Trichosurus vulpecula*) in New Zealand, and the European wild boar (*Sus scrofa*) in the Iberian Peninsula represent wildlife reservoirs of *M. bovis* and can be a persistent source of re-infection of cattle (5–12). Attempts to control or eliminate these reservoirs of infection have involved population reductions through hunting, trapping or poisoning, as well as physical exclusion of wildlife from cattle feeding areas through barrier fencing. In all cases, vaccination of wildlife to reduce wildlife-to-cattle transmission has been investigated, with some vaccines progressing to field trials (13, 14). The goal of vaccination is to induce an immune response such that the animal is resistant to infection or if infection occurs, disease severity is lessened and transmission is reduced or eliminated. Thus, a successful wildlife vaccine need not provide complete protection from infection (15, 16).

Vaccines other than BCG have been successfully used in wildlife to control rabies in raccoons (*Procyon lotor*), foxes (*Vulpes vulpes*), skunks (*Mephitis mephitis*) and coyotes (*Canis latrans*) in Europe and North America (17–19); plague in North American black-tailed prairie dogs (*Cynomys ludovicianus*) (20–23); and classical swine fever in wild boar (*Sus scrofa*) in Europe (24). There have been no widespread efforts to vaccinate wildlife to control tuberculosis, although there is currently one approved vaccine for use in European badgers (25) and field trials are progressing (14).

## History of BCG

The most studied tuberculosis vaccine in deer, as well as other wildlife is the attenuated strain of *M. bovis* known as bacillus Calmette-Guérin (BCG), named for Albert Calmette and Camille Guérin, two French scientists at the Pasteur Institute that developed the strain (26). BCG vaccines are the oldest vaccines still in use today; moreover, with over four billion people vaccinated in over 180 countries it is history's safest and most widely used vaccine (27) and it remains the only approved tuberculosis vaccine for humans. Protective immunity in adults is highly variable, ranging from 0 to 80% depending on the study (28). In adults, BCG vaccination does not reliably prevent infection, development of latent tuberculosis, or reactivation of latent disease (29). However, in infants BCG has proven beneficial and highly cost-effective in protecting children from tuberculous meningitis (30, 31).

In 1901, French veterinarian and microbiologist, Edmond Nocard transferred to Calmette and Guérin a virulent isolate of *M. bovis* he had recovered from a cow with tuberculous mastitis (32). From this isolate, BCG was developed through continuous subculture on a media composed of ox bile, glycerin and potatoes. In 1919, after 13 years and 231 subculture passages, virulence in various animal models was lost (i.e., rabbits, guinea pigs, cows, horses, hamsters, mice, dogs, chickens, non-human primates) (33–35). The attenuation of BCG was shown to be irreversible upon further cultivation on bile-potato medium (36) and passage through various animal species (33). The first human was vaccinated in 1921 when an infant was orally dosed with live BCG. The infant's mother had died of tuberculosis and the infant's caregiver, the grandmother, had clinical tuberculosis. In spite of what must have been significant exposure to virulent *M. tuberculosis*, the child developed normally with no signs of tuberculosis (33). In the following 3 months after this first vaccination, 317 infants were vaccinated and by 1924 more than 660 infants had been orally vaccinated (26). Oral, subcutaneous, intraperitoneal and intravenous routes of administration all proved safe. Although originally given orally, the current recommendation for BCG vaccination is intradermal injection (37). The original BCG was not cloned, but was distributed to many laboratories worldwide, where the vaccine was propagated, such that today there are many genetically variant BCG strains, none of which are identical to each other or to the original vaccine (26, 32, 38). The various substrains differ in immunogenicity and potency; a possible reason for historically large ranges of observed efficacy in human studies around the world (26, 32, 39). Currently, five strains account for >90% of the BCG used worldwide; Pasteur 1173 P2, Danish 1331, Glaxo 1077, Tokyo 172-1, Russian BCG-I and Moreau RDJ (40). The two strains most commonly used in deer studies are strains Danish 1331 and Pasteur 1173 P2. The isolate that would later become BCG Danish was received directly from Calmette in 1931 by Statens Serum Institut. In 1960, batch 1331 was freeze-dried and eventually adopted as the primary Danish 1331 seed-lot in 1966 (32). The strain Pasteur 1173 P2 originated in 1961; produced from a colony closely resembling the original descriptions of BCG by Calmette (32). In white-tailed deer studies, both strains have demonstrated some degree of protection (41).

Calmette and Guérin recognized in animal studies that vaccination prevented disease, but did not always prevent infection (36), a finding consistent with most modern BCG studies in animals (42–45). Although developed as a vaccine for humans, it was first proven efficacious in cattle circa 1911. Calmette and Guérin recommended widespread oral BCG vaccination of neonatal calves, since older calves may have already been infected with virulent *M. bovis* (36). Safety studies in other mammals including horses, sheep, dogs, rabbits, guinea pigs, non-human primates, rats, mice, chickens, and pigeons showed no untoward effects (33).

## Model of infection

To study vaccine-induced protection, a reliable model of infection is of paramount importance. The ideal model is repeatable, technically feasible, and produces disease similar to that seen in natural infection. The best and most widely used model of tuberculosis in deer was developed in New Zealand using red deer (*Cervus elaphus*) and a low dose (200–500 colony forming units, CFU) intratonsilar inoculation (46); where virulent *M. bovis* is deposited into one or both palatine tonsillar crypts. Using this model, many experiments were carried out to identify critical variables in BCG studies, such as dose, route, boosting and detailed immune responses (47–52). The red deer model has been extended for use in white-tailed deer (53). In both deer species, the intratonsilar model results in primary involvement of the medial retropharyngeal lymph node (46, 53), the most commonly affected tissue in naturally infected deer (54–56). The frequent involvement of the medial retropharyngeal lymph node suggests that the primary route of infection in deer is oral; although contribution by aerosol cannot be excluded (57–59). Further supporting a primary oral route of infection is the finding that experimental infection of white-tailed deer via an aerosol did not result in lesion distribution similar to natural infection, but rather resulted in disease focused on the lungs and pulmonary lymph nodes (60).

## Vaccine efficacy

Vaccine doses of 10^4^-10^7^ CFU of BCG provided significant levels of protection against infection and disease (lesion development) in red deer (51), while 10^7^ CFU (parenteral) and 10^8^ CFU (oral) demonstrated similar efficacy in white-tailed deer (41, 61, 62).

There are no known antemortem immune responses that correlate to BCG-induced protection. Measurements of immune responses to vaccination such as intradermal skin testing or cytokine production do not predict protection in any species. Rather, BCG efficacy is measured through postmortem quantitative or semi quantitative assessments of disease severity, as well as measuring the level of tissue colonization (63, 64). Disease severity assessments include subjective scoring of gross lesions based on size, number, presence of liquefactive or caseous necrosis or fibrous encapsulation, and the number of tissues with lesions and from which virulent *M. bovis* can be isolated. Protection has also been evaluated by considering the extent and distribution of lesions, that is, animals with lesions limited to a single body region are considered more protected than those with lesions in multiple anatomic locations such as cranial lymph nodes, thoracic lymph nodes and abdominal organs (41, 43, 61, 62, 65).

In white-tailed deer and red deer, oral (43, 51, 62) or subcutaneous (41, 43, 51, 61) BCG vaccination results in fewer lesions, as well as fewer tissues from which virulent *M. bovis* may be isolated. Using subjective gross lesion scoring, BCG vaccination of deer decreases lesion severity and limits disease dissemination. Microscopic examination of tissues reveals that vaccinated deer have fewer large necrotic lesions that contain large numbers of acid-fast bacilli compared to non-vaccinated animals (41, 43, 61, 62). Both live and inactivated BCG in saline and oil adjuvant, as well as a recombinant BCG expressing the inflammatory cytokine IL-2 have been evaluated in red deer (66, 67). Detailed studies show significant immune responses to some of these preparations; however, necropsy and pathology results are not always available from these studies making vaccine efficacy determination difficult. Studies in red deer have also shown that a homologous prime boost regime (i.e., two doses 4–8-weeks apart), further reduces infection and disease (48, 68, 69). A single study in white-tailed deer demonstrated no significant difference between a single vaccination and a homologous prime-boost approach (61). Reduction of disease transmission through BCG vaccination remains to be demonstrated in deer.

In other wildlife species, the time to seroconversion, and transmission from adults to offspring have been used to demonstrate BCG-induced protection in European badgers (13, 14). The median time to seroconversion was significantly longer for vaccinated badgers (413 days), compared to non-vaccinates (230 days) (14). In addition to a direct protective effect of badger vaccination, there was a positive indirect effect on unvaccinated badger cubs. When at least one third of a badger social group was BCG vaccinated, the probability of an unvaccinated badger cub testing positive for *M. bovis* infection was reduced by 79% (13). The use of such metrics in deer would be difficult due to differing social structures, fecundity and biology.

## Vaccine delivery

The most efficacious vaccine is of little use if it cannot be delivered to the target population. An effective means of delivery requires knowledge of host feeding behavior, climatic effects on bait matrix composition, environmental survivability of the vaccine, and bait attractiveness and palatability to the target host. In most cases the only effective means to vaccinate wildlife is through an oral bait. Oral vaccines have been used experimentally to protect white-tailed deer from the prion-based, chronic wasting disease (70, 71), as well as brucellosis (72).

A variety of oral baits have been evaluated in wildlife. Dried shell corn has been used to deliver an acaricide to free-ranging white-tailed deer (73, 74) while Hakim, et al showed that free-ranging white-tailed deer found a liquid bait composed of apple juice, water and glycerin palatable; thus a plausible means of delivering pharmaceutical agents (75). A molasses-based bait for potential BCG delivery was evaluated for palatability, attractiveness and stability under various environmental conditions (76). Although environmentally stable and attractive for captive deer, field testing demonstrated a lack of palatability to free-ranging deer. A lipid formulation of BCG has been used as an oral vaccine for brushtail possums (77, 78), and European badgers (45, 79). The same BCG lipid-formulated bait has been used in white-tailed deer, and although vaccination was achievable (43, 80), deer found the lipid formulation unpalatable. In Spain, baits prepared from feed mixed with paraffin, sucrose and cinnamon-truffle powder worked well to deliver BCG to wild boar (81, 82), but have not been evaluated in deer.

A potential hazard of oral bait vaccines, is the difficulty of preventing non-target species from consuming the vaccine bait. Cattle are a non-target species of special interest as it is possible that BCG ingestion could result in sensitization to the tuberculin used in intradermal skin testing resulting in false positive results; thus, confounding accurate identification of infected cattle (83). Alternative diagnostic tests, able to differentiate infected from vaccinated (DIVA) cattle would be needed to avoid this confounding problem (84–86). In addition to exposure of non-target species to vaccine, dosage is difficult to control using oral baits. The effect of higher than recommended doses of vaccine should be evaluated in the target population. In red deer, no untoward effects have been seen using subcutaneous doses of BCG up to 1 × 10^8^ CFU (68); 10–100 times the regular dose, or in white-tailed deer using oral doses of 1 × 10^9^ (80, 87) to 1 × 10^10^ CFU; 10–100 times the regular dose.

Studies in red deer did not demonstrate shedding of BCG from vaccinates to non-vaccinates (66); however, evidence shows that BCG-vaccinated white-tailed deer shed vaccine and cohorts can become “secondarily vaccinated” (88, 89). It remains to be evaluated whether deer vaccinated secondarily through shed BCG possess any protection against infection with virulent *M. bovis*. If secondary vaccination were to provide protection, this self-disseminating feature could serve to increase vaccine coverage without additional labor or cost. However, the shedding of BCG by deer increases the possibility that non-target species such as cattle could be exposed to BCG. Thus far, indirect contact of calves with BCG-vaccinated white-tailed deer has not resulted in deer-to-cattle transfer of BCG (88, 89).

By comparison, orally vaccinated possums and badgers were shown to shed BCG in feces for up to 7 and 17 days, respectively, after vaccination (44, 90), while excretion could not be detected in orally vaccinated wild boar (82).

## Safety

Vaccine safety may be viewed from both the perspective of either the vaccinated animal or humans that may come into contact with vaccinated animals. No untoward effects have been reported in BCG-vaccinated deer, possums or badgers (66, 91, 92). In white-tailed deer vaccinated subcutaneously with BCG, but not challenged with virulent *M. bovis*, microscopic, but not gross lesions due to BCG were reported in various lymph nodes (superficial cervical, tracheobronchial, hepatic) as late as 250 days after vaccination (41).

Although BCG has proven safe in humans with uncompromised immune systems, use of BCG in immunocompromised individuals can result in disseminated disease, with infection in various organs and body systems (93, 94). Because BCG may persist in tissues of vaccinated deer, hunters could potentially be exposed to BCG while field dressing vaccinated deer and unlike many other wildlife hosts of *M. bovis*, deer may be consumed as food by humans. In BCG-vaccinated white-tailed deer, vaccine was recovered from lymphoid tissues up to 12 months after oral dosing of 10^9^ CFU. Lowering the dose to 10^8^ CFU decreased persistence to 9 months. Persistent and viable BCG were limited to lymphoid tissues such as cranial lymph nodes, tracheobronchial, hepatic and mesenteric lymph nodes. Importantly, samples of muscles commonly consumed by hunters (epaxial, sublumbar, supraspinatus, triceps, semimembranosus, semitendinosus and biceps femoris) did not yield viable BCG at any time point (80, 87). In BCG-vaccinated red deer, viable vaccine could be recovered from various lymph nodes and the site of vaccination 14 weeks after vaccination, although the numbers of recoverable CFU were extremely low, 32–57 CFU/node and 150–190 CFU/vaccination site, representing 0.007–0.009% and 0.002–0.003%, respectively, of the original inoculum dose (2 × 10^6^ CFU) (67). It has been shown that thoroughly heating meat products to 60°C (140^0^ F) for 6 min kills virulent *M. bovis* (95) and *M. avium* (96). It is assumed the same would be true for *M. bovis* BCG. As humans generally avoid consumption of lymphoid organs and usually cook meat before consumption (97), the potential exposure of humans to BCG from vaccinated deer is very low.

By comparison, BCG has been found in the tissues of orally vaccinated badgers 30 weeks after vaccination (44) and in possums 8 weeks after oral vaccination (90). In contrast, BCG could not be found in the tissues of orally vaccinated wild boar (82) even when examined 30 days after vaccination (98), an important finding as wild boar, similar to deer, are often used for food.

## Non-tuberculous mycobacteria

Many saprophytic, non-pathogenic species of mycobacteria exist in soil and water. These mycobacteria may be collectively described as non-tuberculous mycobacteria (NTM). Numerous NTM have been isolated from deer (41, 61, 62, 80, 99–101), some of which were found within lesions consistent with tuberculosis. Although some studies have suggested that preexisting sensitivities to *M.avium*, or other NTM, has no effect or confers some degree of protection against virulent challenge (102–106), others show interference with BCG efficacy by NTM exposure in humans, laboratory animals and cattle (102, 104, 107, 108). One proposed mechanism for this reduced efficacy is that pre-existing immune sensitivity to NTM restricts BCG multiplication following vaccination, resulting in dampening of critical cytokine responses, such as that of interferon-gamma (108). For this reason, it is recommended that humans and calves be vaccinated as neonates prior to NTM exposure. It is, as yet unclear how exposure to NTM affects BCG efficacy in deer. Vaccination of neonates, although possible in farmed deer, would prove very difficult in free ranging deer.

## Future directions

### Self-disseminating virus-based vaccines

One limitation of traditional oral or parenteral vaccination is the need to administer vaccine to every animal individually. Furthermore, with many inactivated vaccines, adequate protection requires subsequent booster vaccinations. In contrast, self-disseminating vaccines are designed to exploit replicating virus-based vectors to spread within the target animal population without the need for individual animal inoculation (109). Vaccination of a limited number of animals introduces the vaccine into the target population and the vaccine is spread naturally as it is shed by vaccinates. Ideal self-disseminating vaccines are viruses with high immunogenicity and high horizontal transmission levels, but with a robust species barrier to minimize infection of non-target species. Examples of self-disseminating virus-based vaccines include a cytomegalovirus-based vaccine targeting deer mice (*Peromyscus maniculatus*) to interrupt transmission of Sin Nombre hantavirus, and a myxoma virus-based vaccine targeting European hares (*Oryctolagus cuniculus*) to prevent myxomatosis and rabbit hemorrhagic disease [reviewed in Murphy et al. (109)]. A similar self-disseminating viral vectored vaccine targeting white-tailed deer to prevent deer-to-deer and deer-to-cattle transmission of *M. bovis* may one day be possible.

### Plant-based vaccines

Another alternative to traditional vaccination is the use of plant-based vaccines (110). Selected immunogenic antigens of the pathogen are introduced into a plant, creating a recombinant edible vaccine. Ingestion of the plant material induces a protective immune response against that particular pathogen. Plant-based vaccines are cost-effective and amenable to large scale production (110); moreover, using plants that are part of the normal diet of the target population minimizes issues of palatability and acceptance. Edible vaccines have been produced in tobacco, cereal grains, fruits (banana, tomato), leaves (lettuce, alfalfa), tubers (potato, carrot), and legumes (cow pea, soybean) (111). When produced in plants, antigenic proteins of the vaccine are bioencapsulated in plant cells, to be released when plant cells are digested by microbes of the gut (112). This may be particularly advantageous with diseases such as tuberculosis where mucosal immune responses are critical. Transgenic carrots, tobacco, lettuce and arabidopsis expressing *M. tuberculosis* proteins have been tested in mice and piglets and shown to induce both humoral and cell-mediated immunity (112–115).

### Inactivated vaccines

Attenuated live vaccines, like BCG have some drawbacks. The possibility exists that vaccine shed by vaccinates, may contaminate not only the environment, but also potentially expose various non-target species. Use of genetically altered subunit vaccines may be an alternative; however, there could be public resistance to the use of genetically altered microbes. Heat-inactivated *M. bovis* (oral and parenteral) has been shown to reduce disease severity in wild boar (82, 116) similar to protection provided through vaccination with BCG (117), without risk of environmental contamination or spread to non-target species. Similarly, heat-inactivated *M. bovis* has been shown to decrease disease severity in experimentally infected red deer (118). Another noted advantage to heat-inactivated *M. bovis* is that vaccinated calves did not have false positive responses in either antibody-based assays or interferon gamma release assays measuring cell-mediated immune responses (118) reducing concern that vaccine exposed cattle would be falsely identified as *M. bovis* infected during routine surveillance.

## Conclusions

Between 1940 and 2004, more than 335 emerging infectious disease events were reported in the scientific literature. The majority (60%) of those events involved zoonoses, most of which (72–80%) had an epidemiologically important wildlife host (119, 120). Controlling or eliminating disease, which has become established in wildlife is extremely difficult, with seemingly few solutions, such as population reduction, separation of wildlife from livestock and disease control through vaccination. Varying degrees of success have been achieved with rabies, plague and classical swine fever. In the case of tuberculosis in deer and other wildlife, the challenge is indeed monumental. In spite of millions of research dollars and countless hours of research effort toward a new human vaccine, the only approved vaccine remains one that is 100 years old and provides questionable protection in some settings. Far less money and effort have been expended exploring a vaccine for animal tuberculosis. Nevertheless, there is reason to be optimistic. Regardless of the species, research to date on BCG vaccination consistently demonstrates a decrease in disease severity, which likely results in decreased disease transmission, and progress is being made in the development of oral baits as vaccine delivery devices. Moreover, advances are being made in the next-generation of human vaccines based on BCG (79), some of which may prove useful for vaccination of deer or other wildlife.

## Author contributions

All authors listed have made a substantial, direct and intellectual contribution to the work, and approved it for publication.

### Conflict of interest statement

The authors declare that the research was conducted in the absence of any commercial or financial relationships that could be construed as a potential conflict of interest.
